# Management of COVID-19 Patients in the Emergency Department

**DOI:** 10.3390/jpm11100961

**Published:** 2021-09-27

**Authors:** Ioannis Pantazopoulos, Stamatoula Tsikrika, Stavroula Kolokytha, Emmanouil Manos, Konstantinos Porpodis

**Affiliations:** 1 Department of Emergency Medicine, Faculty of Medicine, University of Thessaly, Biopolis, 415 00 Larissa, Greece; 2 Emergency Department, Thoracic Diseases COVID-19 Referral Hospital “SOTIRIA”, 115 27 Athens, Greece; matatsik@yahoo.gr; 3 Department of Emergency Medicine, Sismanoglio Hospital, 151 26 Athens, Greece; st_kolokytha@hotmail.com; 4 Pulmonary Clinic, General Hospital of Lamia, 351 00 Lamia, Greece; emanuilmanos@hotmail.com; 5 Respiratory Medicine Department, Aristotle University of Thessaloniki, G Papanikolaou Hospital, 570 10 Thessaloniki, Greece; kporpodis@yahoo.gr

**Keywords:** emergency department, emergency physicians, COVID-19, SARS-CoV-2

## Abstract

COVID-19 is an emerging disease of global public health concern. As the pandemic overwhelmed emergency departments (EDs), a restructuring of emergency care delivery became necessary in many hospitals. Furthermore, with more than 2000 papers being published each week, keeping up with ever-changing information has proven to be difficult for emergency physicians. The aim of the present review is to provide emergency physician with a summary of the current literature regarding the management of COVID-19 patients in the emergency department.

## 1. Introduction

COVID-19 (coronavirus disease 2019) is the disease resulting from infection by the SARS-CoV-2 (severe acute respiratory syndrome coronavirus 2) virus. First identified in Wuhan, China, on 31 December 2019, the disease rapidly developed into a global pandemic. On 26 February 2020, the first COVID-19 case was reported in Greece and by 26 June 2021, more than 12,500 patients had already died [1]. As the pandemic overwhelmed emergency departments (EDs), a restructuring of emergency care delivery became necessary in many hospitals. Furthermore, with more than 2000 papers being published each week, keeping up with ever-changing information has proven to be difficult for emergency physicians [2].

The aim of the present review is to provide emergency physician with a summary of the current literature regarding the management of COVID-19 patients in the emergency department.

## 2. Triage of Suspected COVID-19 Patients

**Figure 1:**
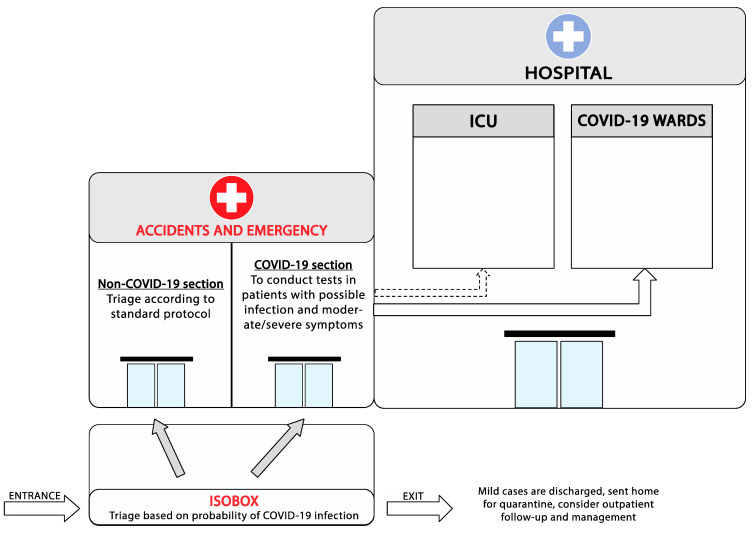
Emergency department reorganization for the management of COVID-19 outbreak.

Health professionals performing triage process should promptly screen patients for possible COVID-19 symptoms and simultaneously manage the risk classification for disease severity ideally in a prehospital isobox facility (Figure 1). If a space does not exist for an isobox, then even a well-ventilated room inside the built structure or a well signaled tent outside could be the solution for small healthcare facilities. Additionally, in the triage area, a uni-directional flow of patients in one-way in and one-way out, an emergency isolation pathway for critically ill patients, available COVID-19 testing for early evaluation in ER area, and a secured connection into an intensive care unit (ICU) or advanced healthcare care and resources, should be obtained [3,4]. Dedicated clinical staff should be assigned for the evaluation of patients presenting with COVID-19 symptoms at triage. These staff should be trained on triage procedures, COVID-19 case definition, and appropriate personal protective equipment use (i.e., mask, eye protection, gown, and gloves) [5]. The priority is to minimize virus transmission, reduce lengthy waiting times, and enhance the overall outcome among patients with COVID-19 disease [6]. As disease severity may vary from asymptomatic to severe acute respiratory distress syndrome (ARDS), the severity of illness should be identified among with features that recognize patients that are most at risk for clinical deterioration [7]. Critically ill patients should be transferred to the ICU as soon as possible with no delay while patients who require lab and imaging tests should be admitted to the ED [8].

All suspected and confirmed cases admitted to the ED for further testing should be managed with effective isolation and protective measures. Healthcare facilities without enough single isolation rooms or those located in areas with high community transmission should designate a separate, well-ventilated area where patients at high risk for COVID-19 can wait. This area should have benches, stalls, or chairs separated by at least one-meter distance. The waiting areas should have dedicated toilets and hand hygiene stations. Patients who are suspected to have COVID-19 should not be mixed with COVID-19 confirmed patients in isolation areas [9].

Moreover, obvious signs in each triage station or waiting areas with precautions measures such as appropriate physical distancing between patients, hand hygiene, and respiratory hygiene should be applied. A limited number of family members and caregivers can accompany patients who need assistance and must always be in accordance with COVID-19 national guidelines and local protocols. Each healthcare facility should follow previously agreed local or regional preventive protocols against contamination of COVID-19 in the ED, including screening for COVID-19 among triage patients [4,10].

## 3. Severity of Illness at ED Presentation

Adults with SARS-CoV-2 may present to the ED with a wide range of clinical manifestations according to illness severity. The National Institute of Health (NIH) reports that patients who have any signs and symptoms of COVID-19 (e.g., fever, cough, sore throat, malaise, headache, muscle pain, nausea, vomiting, diarrhea, loss of taste or smell) but who do not have dyspnea, or abnormal chest imaging have mild illness. Most of them will be managed in an ambulatory setting or at home, e.g., using telemedicine. Individuals with evidence of lower respiratory disease during clinical assessment or imaging with an oxygen saturation (SpO_2_) ≥ 94% on room air have moderate illness. Patients with moderate disease should be admitted and closely monitored, because the progress of COVID-19 can be abrupt, and the clinical condition may deteriorate rapidly. Severe illness is diagnosed in patients with SpO_2_ < 94% on room air, a ratio of arterial partial pressure of oxygen to fraction of inspired oxygen (PaO_2_/FiO_2_) < 300 mm Hg, respiratory frequency >30 breaths/min, or lung infiltrates > 50%. These patients will require supportive oxygen therapy and hospital admission. Finally, patients with respiratory failure, septic shock, and/or multiple organ dysfunction are in a critical condition and must be admitted to the ICU [11].

## 4. Predicting Deterioration Risk

Triaging patients at admission to determine subsequent deterioration risk (defined as any requirement of ventilatory support or ICU admission or death) is difficult, especially in COVID-19 patients, since many of them develop early lung injury and hypoxia before clinical deterioration is appreciated [12]. However, effective triage is crucial for informing clinical decision making and facilitating resource allocation. Several tests have been proposed for COVID-19 patients and have been examined as triaging tools for in-hospital clinical deterioration, such as the Rothman Index, a well-established acuity measure score, the quick Sequential (Sepsis-related) Organ Failure Assessment (SOFA) score that identifies non-ICU patients with suspected infection who are at a high risk for in-hospital mortality, the CURB-65 or CRB-65 and the A-DROP (a modified CURB-65 score) that estimate the mortality of community acquired pneumonia, the National Early Warning Score (NEWS) score that determines the degree of illness of a patient and prompts critical care intervention, the Modified Early Warning Score (MEWS) that identifies patients who are at risk for clinical deterioration and who may require a higher level of care, the Rapid Emergency Medicine Score (REMS) that predicts in-hospital mortality in nonsurgical patients presenting to the ED, and the pneumonia severity index (PSI) that calculates the probability of morbidity and mortality among patients with community acquired pneumonia (Table 1) [13,14,15,16,17,18]. However, none of these tests have reached sufficient performance and cannot be used in clinical decision-making in COVID-19 patients.

Of note, researchers from the United Kingdom developed the 4C (Coronavirus Clinical Characterisation Consortium) Mortality Score which uses readily available data, such as age, sex, number of comorbidities, respiratory rate, oxygen level, level of consciousness, urea, and C-reactive protein, to accurately categorize patients at low, intermediate, high, or very high risk of death [19]. Patients at low risk may be suitable for management in the community, while those at intermediate risk may need admission and ward level monitoring. Patients at high risk will usually need aggressive treatment including early escalation to critical care if necessary. The 4C Mortality Score is already recommended by the NHS for use in the United Kingdom to guide antiviral treatments.

In addition, researchers from the International Severe Acute Respiratory and Emerging Infections Consortium Coronavirus Clinical Characterisation Consortium (ISARIC4C) developed a risk stratification tool called the “4C Deterioration Score”. This score assesses 11 parameters that can be routinely collected from patients, including age, gender, and physical measurements (such as oxygen levels), along with some standard laboratory tests, and calculates the risk of deterioration. This tool was developed using data from 74,944 patients with COVID-19 who were admitted to 260 hospitals across England, Scotland, and Wales, and has shown superior performance in comparison to previous risk scores [20]. The addition of the 4C Deterioration Score along with the 4C Mortality Score provides an evidence-based method to identify those who will need aggressive support during admission, even if they have a low risk of death. Both scores are very promising and could be used widely after a careful evaluation of their accuracy.

## 5. Correlation of Imaging Findings with Prognosis

Patients with COVID-19 and normal or near normal chest X-rays typically have a benign clinical course [21], while severely ill patients usually have bilateral findings in more than one lobe. Chest X-rays triage for pneumonia in non-severe COVID-19 patients in the ED can be an effective strategy to optimize resource use [22].

The most common chest computed tomography (CT) findings in patients with COVID-19 infection are consolidations and/or ground-glass opacities, which are very often bilateral, peripheral, and located primarily in the lower lobes [23,24,25,26]. The chest CT findings play a key role not only in the diagnosis of COVID-19 but also in the monitoring of disease progression [25]. The correlation between CT findings and disease severity has been demonstrated in many studies [26]. Bilateral and multilobar infiltrations on initial chest CT are associated with poor prognosis, with the presence of ARDS being the most common indication for ICU transfer [26].

## 6. Indications for Chest CT in the ED

Chest CT is not routinely recommended in patients with mild symptoms unless they are at risk for disease progression [25,27]. It is also not recommended as a routine procedure in asymptomatic patients. Chest CT is recommended in patients with moderate or severe disease and in those with features of respiratory deterioration [25]. CT allows for the identification of signs of pulmonary edema, raising the suspicion of COVID-19 related myocarditis, in which case troponin measurement and ECG may be required [27]. Finally, an indication for CT angiography is the suspicion of pulmonary embolism in patients with COVID-19 infection with limited disease extension if supplementary oxygen is needed or in those with clinical criteria of severity with an elevated D-dimer level and the absence of any anticoagulant therapy [27,28].

## 7. The Role of Lung Ultrasound in COVID-19 Disease

Although chest CT is the standard imaging modality in early diagnosis and management of COVID-19, the use of lung ultrasound (US) presents some advantages (radiation-free, flexibility, and cost-effective) over the use of chest CT and may play a complementary role in the workup of COVID-19 [29,30]. In the ED, lung US can be employed for an early detection of pulmonary involvement in symptomatic patients suspected for COVID-19, with still pending real-time Reverse Transcription Polymerase Chain Reaction (rRT–PCR), as soon as they arrive [31]. It can also be used in several moments of the natural history of the SARS-CoV-2 (colonization/infection) as it can identify the pulmonary involvement and seriousness of the disease in patients with suspected or confirmed COVID-19 [32]. It rapidly identifies pulmonary involvement and provides risk stratification, including a prediction of the need for mechanical ventilation and mortality [33].

Lung US shows a sensitivity and specificity of 85–93% and 86–93%, respectively, in the diagnosis of pneumonia [34]. COVID-19 causes clear and typical ultrasonographic patterns. B lines occur in large numbers, both in separate and coalescent forms (light-beam patterns) and can give the appearance of a shining white lung. Irregularity of the pleural line, sub-pleural pulmonary consolidations, and poor blood flow also occur in bilateral patchy clusters and are mainly visible in the posterior and inferior areas [35]. The composition of different density of B-lines and areas of consolidation show parallel changes with the clinical severity. The extent of disease demonstrated by US findings seems to reach the peak at the second week, recovering gradually thereafter [30]. On the other hand, thickening of the pleural line in the inferior and posterolateral sites is indicative of pneumonia or ARDS [36]. There is also a strong correlation between similar US findings and concurrent CT scans [37]. A potential use of lung US could be in patients who are swab PCR negative and have an indeterminate chest X-ray: the presence of the above classical changes of COVID-19 would suggest a false negative swab result and allow a firm diagnosis to be established [35]. A typical lung US examination should lead to managing the patient as having a high probability for COVID-19, and to repeating the swab [31]. Although lung US is unlikely to replace PCR as a confirmatory test, it has the potential to be faster, repeatable, and to contribute additional clinical information at the time of care [38]. Early recognition of the lung involvement of COVID-19 is especially important because lung involvement seems to play a critical role in the development of pneumonia, ARDS, and multiorgan failure [39]. When combined with focused cardiac ultrasound, it may be useful in identifying alternative pathologies resulting in respiratory failure as well [38]. Lung US may also be considered as an alternative imaging method for pregnant women, as it is radiation-free and can be safely used multiple times for serial examinations [40]. In conclusion, lung US could serve as a valuable tool for the detection and follow-up of lung lesions in COVID-19 pneumonia and also provide supplemental imaging information for currently recommended radiological examinations, with the advantages of being radiation-free, flexibility, and cost effective [30], as an alternative to chest CT. Although lung US cannot replace CT, which is the gold standard for lung evaluation, lung US may be considered a reliable tool in pregnancy [41].

## 8. Lab Tests Associated with Worse Prognosis

Asymptomatic patients or those with mild disease should not routinely undergo laboratory blood tests unless it is otherwise indicated at the discretion of each physician. Patients treated in the ED with moderate, severe, or critical COVID-19 infection are recommended to be tested with complete blood count (white blood cell-type, platelets), biochemical tests (urea, creatinine, liver enzymes, albumin, creatine phosphokinase, lactate dehydrogenase), coagulation markers (d-dimers, prothrombin time), and inflammatory markers (ferritin, C-reactive protein, procalcitonin) [42]. Worse disease outcome is associated with the presence of thrombocytopenia (<100,000–150,000/mm^3^) or neutropenia (<800–1000/μL) [43,44,45], the presence of new acute kidney injury and elevated transaminases, troponin, lactate dehydrogenase (>250 U/L), d-dimers, and CRP (>100 mg/L) [44,46]. It has been found that the percentage of lymphocytes is inversely associated with the severity of the disease and the presence of lymphopenia is associated with a three-fold increased risk of severe disease [47]. Lymphopenia is also associated with severe disease, especially in young patients [43]. Regarding d-dimers, a two-fold increase in values has consistently been shown to predict disease severity in numerous studies. A recent meta-analysis demonstrated a moderate accuracy in predicting severe and fatal cases of COVID-19 (sensitivity 77% and 75% and specificity 71% and 83% for predicting severity and mortality, respectively) and a high sensitivity (90%) but relatively low specificity (60%) for detecting venous thromboembolism (VTE) [48]. Thus, d-dimers can be used as both risk stratification and screening tool in patients with COVID-19. On the other hand, mild elevations in cardiac troponin concentrations, especially in older patients, may reflect a pre-existing cardiac disease e.g., coronary heart disease, atrial fibrillation, and acute myocardial injury related to COVID-19 infection or pneumonia [49]. Moreover, ferritin levels greater than 300 ng/mL have been associated with in-hospital mortality at an odds ratio of 9.10 [50]. Finally, checking creatine phosphokinase levels in patients with significant myalgias may help in the identification of patients with COVID-19 related myositis [51].

A promising prognostic biomarker called soluble urokinase plasminogen activator receptor (suPAR) is currently used across various hospitals in Europe to manage COVID-19 patients. suPAR is a protein in the blood that reflects immune activation [52]. It has been demonstrated that the admission suPAR level is an early indicator for the risk of developing severe respiratory failure and requiring mechanical ventilation [53]. Using admission suPAR levels for COVID-19 patients, physicians may identify low-risk patients for early discharge to reduce the pressure on COVID-19 emergency departments. Furthermore, physicians may identify high-risk patients for early treatment [52]. A recent study by Rovina et al. evaluated whether the suPAR level at the time of admission could identify patients who would likely develop severe respiratory failure within the first 14 days [53]. It appeared that a suPAR-level ≥ 6 ng/mL, a cut-off point identified using ROC-analysis, was a strong predictor for developing severe respiratory failure and requiring ventilation within a relatively short period [53]. This was confirmed in an international multicenter study showing that very few COVID-19 patients with suPAR below 4.6 ng/mL developed respiratory failure (N = 3, 2.6%) compared to patients with suPAR above 6.86 ng/mL (N = 53, 44.9%) [54]. Furthermore, it has been demonstrated that the suPAR level at the time of admission was a strong predictor of developing in-hospital acute kidney injury and the need for dialysis. The higher the suPAR-level, the more severe the outcome. For patients admitted with a suPAR-level < 4.60 ng/mL, only a 6.0% incidence of AKI was found with no patients developing a need for dialysis [54]. For patients admitted with a suPAR level > 6.86 ng/mL, there was a 45.8% incidence of AKI with 16.1% of those patients requiring dialysis [54].

## 9. Confirmation of SARS-CoV-2 in the ED

In addition to clinical diagnostic criteria including symptoms, laboratory markers, and imaging tests, diagnostic approaches to COVID-19 pandemic heretofore require determined laboratory testing strategies, principally Nucleic Acid Amplification Tests (NAATs), such as rRT-PCR targeting viral genes, antibody tests, and Rapid Diagnostic Tests (RDTs), including Antigen RDTs and Immunoglubin RDTs.

Generally, NAATs are believed to be the most sensitive methods for a pathogen detection [55,56] whereas RDTs are recommended by the World Health Organization (WHO) mainly in research and low-income countries [57]. RT-PCR is recommended as the most sensitive NAAT method [55,56]. Like all diagnostic tests, rRT-PCR is not completely foolproof and false-negativity has been reported. False negatives have been reported to occur in ∼30% (range 10–40%) of patients with COVID-19 [56,58]. Multiple factors may contribute to false RT-PCR results, including: (1) a high limit of detection score (LoD score) in a specific RT-PCR kit; (2) sample collection when the viral load is low (e.g., early after exposure and before the peak associated with symptom onset, or late in disease course); (3) faulty sample collection technique especially from unqualified personnel resulting in reduced viral specimens and load; (4) sample degradation from inadequate preservation, delays in transportation, and processing of the unstable RNA virus, as specimens may degrade without appropriate transport medium or storage conditions; (5) specific SARS-CoV-2 RNA mutations that escape detection; (6) RT-PCR inhibitors in the sample (bloody or viscous sample, such as in several respiratory preexisting medical conditions); (7) technical limitations of the RT-PCR kit’s interim guidance [57,58]. One pooled analysis found the probability of a false-negative result ranged from 100% on day 1 after infection to 21% on day 9 to 66% on day 21 [59]. The type and site of sample collection is crucial for the sensitivity of the assays. The rate of RT-PCR detection of SARS-CoV-2 in patients with COVID-19 is 72% in sputum and 63% in nasopharyngeal swabs, while it is only 32% in pharyngeal swabs [60]. However, only 13% to 30% of patients produce sputum as reported in a recent study under publication [61]. In this study, writers observed a decrease in sensitivity with decreasing disease severity, an increase in sensitivity in immunocompromised patients, and a rapid decline of sensitivity in time post onset of symptoms, but only in outpatients [61].

The rate of RT-PCR detection of SARS-CoV-2 in upper respiratory tract (URT) samples has important consequences for screening, treatment, and isolation measures in hospitals, as precautions may be relaxed in the presence of a negative test, increasing the risk of transmission. Where clinical suspicion and pretest probability is moderate or high, despite an initial negative test of SARS-CoV-2, tests should be repeated, with or without computing tomography scanning, for an accurate diagnosis and subsequent appropriate clinical management and infection control measures. A prompt and precise strategy for managing a false-negative RT-PCR test result, especially in a critical case, is to immediately perform a different sampling (e.g., nasal vs. BAL), or use a different test kit (some laboratories have more test kits with various levels of sensitivity). A combination of two methods, referring to antibody detection combined with genome or antigen tests, is another sophisticated attempt to diagnose a misleading case [57]. In cases where the epidemiological burden in the community is high, the positive predictive value (PPV) of the available tests increases [62].

In the ED of many hospitals worldwide, patients suspected of having COVID-19 are commonly cohorted alongside patients with confirmed diagnosis while awaiting diagnostic test results. This places uninfected patients at excess risk for healthcare-related exposure and transmission. The aforementioned molecular two-step assay usually takes 3.5–4.0 h [62]. Thus, point-of-care (POC) tests are currently being implemented, and they provide results within minutes rather than hours [56]. These may be NAAT, Ag-RDTs, or antibody tests. Most molecular POC tests that are currently used or recently developed demonstrate excellent accuracy with 96–100% PPV and similar negative predictive value (NPV) [56]. In regard to Ag-RDTs, data showed excellent specificity (99.5–100%) and varying overall sensitivity (11.7–68.8%) directly associated with viral loads [56,63]. In a recently published study by Jegerlehner et al., concerning the diagnostic accuracy of a SARS-CoV-2 rapid antigen test in real-life clinical settings, the overall sensitivity of the rapid antigen test was 65.3% (95% confidence interval [CI] 56.8–73.1), the specificity was 99.9% (95% CI 99.5–100.0), while in asymptomatic individuals, the sensitivity was 44.0% (95% CI 24.4–65.1) [64]. Thus, suboptimal sensitivity suggests that Ag testing may be most useful as an adjunct to the gold-standard PCR and negative test results should be treated with great caution, especially in asymptomatic individuals [64]. As a diagnostic tool, antibody serology is useful to detect the presence of antibodies in specific viral antigens within days or weeks following acute infection, for suspected cases that may be missed by NAAT. However, serologic testing should not be used to diagnose acute SARS-CoV-2 infection [65].

Finally, EDs require urgent thought, highly sensitive algorithms, and direct management of all cases, so an emergency physician should avoid test predictions for a false result, and assess each case accurately, based on the sample and all the aforementioned parameters that could have an impact on the final result. Even if ED physicians are not supposed to know laboratory techniques, alternative diagnosis and specific comorbidities such as respiratory issues, should be of first consideration, particularly when clinical suspicion is high.

## 10. Intubation of COVID-19 Patients in the ED

### 10.1. Time of Endotracheal Intubation

The decision for endotracheal intubation and mechanical ventilation in ED patients with severe COVID-19 is challenging because the current guidelines are ambiguous and are not based on strong evidence [66,67,68]. In the absence of strong evidence, the best timing for endotracheal intubation (early vs. late) remains a controversial topic. Most guidelines recommend early intubation of critically ill patients as a means to avoid complications associated with “crash induction” (including cardiac arrest) and protect health care workers from cross-infection [67,68,69,70]. Moreover, many physicians support the notion that early intubation will prevent vigorous respiratory efforts that will increase transpulmonary pressure, leading to patient self-inflicted lung injury (P-SILI), an entity that parallels ventilator induced lung injury (VILI) [71]. On the other hand, the considerations about P-SILI, although very intriguing, are highly speculative and not supported yet by strong evidence as opposed to the well-documented fatal complications of invasive mechanical ventilation [72]. A recent metanalysis reported that the timing of intubation has no effect on the all-cause mortality and morbidity of critically ill patients with COVID-19 and recommended a wait-and-see approach, which may lead to fewer intubations [73]. Based on the available evidence and in the absence of specific indications for tracheal intubation, a trial with nasal high flow or non-invasive ventilation is recommended in critically ill patients with COVID-19.

### 10.2. Indications for Endotracheal Intubation

Indications for endotracheal intubation in patients with COVID-19 include airway protection; severe decompensated acidosis (pH < 7.2–7.25); severe hypoxemia (PaO_2_ < 50 mmHg or SaO_2_ < 90–92%) despite maximal noninvasive respiratory support; clinical signs of increased work of breathing and respiratory muscle fatigue, e.g., use of respiratory accessory muscles, paradoxical motion of the abdomen, and retraction of the intercostal spaces; signs of tissue hypoxia despite maximal noninvasive respiratory support; severe hemodynamic instability; and use of extracorporeal membrane oxygenation (ECMO) [74]. The decision to intubate involves judgement and should be individualized and based not only on the oxygenation status but also the degree of respiratory distress, based on a clinical evaluation of the work of breathing.

The PaO_2_/FiO_2_ ratio is not a reliable marker in non-intubated patients receiving either conventional oxygen therapy or non-invasive ventilatory support because it is often underestimated since the FiO_2_ varies widely due to several factors such as the patient’s ventilatory pattern or the presence of air leaks and it is often overestimated [74]. A low PaO_2_/FiO_2_ ratio, prevention of clinical deterioration, and severity of chest CT findings do not justify per se tracheal intubation [74].

## 11. Initial Ventilator Settings in the ED

Respiratory failure due to COVID-19 has led to debates about when and how to apply invasive mechanical ventilation in these patients. Since the outbreak of the pandemic, approximately 250,000 to half a million people have undergone invasive mechanical ventilation worldwide [75]. We do not have many studies on ventilator management in COVID-19; however, the appropriate management of mechanical ventilation in non-COVID-19 ED patients is associated with improved outcomes [76]. In the early stages of the pandemic, a team of mechanical ventilation experts described two types of COVID-19 respiratory disease, which are, in fact, the extremes of the spectrum of a time-related disease, with some stages and characteristics overlapping. The first is the “L type” pattern, which is found in most patients, characterized by low elastance (i.e, high compliance), low ventilation/perfusion ratio (V/Q ratio), low lung weight, and low recruitability [77]. This phenotype is typical of the early phase of disease, but it can be seen in some severe cases as well.

The other one is the “H type”, the severe form of the disease, which resembles the classic ARDS, characterized by high elastance (i.e., low compliance), high pulmonary shunt, high lung weight, and high recruitability [77]. This phenotype is often seen in the later phase of the disease and patients with this phenotype are usually more severe. As mentioned previously, chest CT is a vital component in not only the diagnostic procedure but also the severity evaluation for COVID-19 patients, with obvious differences in qualitative CT features between the two types. Ground-glass opacities with peripheral lung distribution occur in patients with mild type, while more progressive, organizing, and fibrosis changes, such as consolidation, “crazy paving” pattern, and “white lung” are found in patients with severe type [78].

Gattinoni and colleagues recommended to ventilate “type L” patients, if hypercapnic, with higher tidal volumes (Vt) (7–8 mL/kg of ideal body weight) and lower PEEP (8–10 cmH_2_O) [77]. However, because of the potential for greater ventilator-induced lung injury with higher tidal volumes, it is suggested that clinicians first address common treatable causes of hypercapnia (i.e., inadequate respiratory rate and increased dead space from the ventilator circuit) before resorting to the use of higher tidal volumes [79]. We may liberalize tidal volume (up to 8 mL/kg of predicted body weight) in patients who are double triggering, or if inspiratory airway pressure decreases below PEEP, keeping plateau pressure < 28–30 cm H_2_O and driving pressure below 15 cmH_2_O, although it is suggested that no threshold value exists: the lower the driving pressure, the better the outcome [80]. If compliance is considered to be a marker for disease severity, driving pressure is essentially tidal volume corrected for disease severity. Subsequent studies [75,81,82,83,84] have shown that, on average, COVID-19 related ARDS has similar respiratory system mechanics with ARDS from other causes, so in these patients we should apply the basic principles and evidence-based recommendations for lung protective ventilation [85]. There is significant heterogeneity among patients regarding the possibility of lung recruitability. Not all patients respond in the same way to high PEEP; as such, PEEP settings should be individualized. High PEEP (>12 cmH_2_O) should probably be used in patients with moderate or severe ARDS, but not in patients with mild ARDS [86]. In other words, consider higher PEEP in patients with evidence of a higher potential for recruitment (e.g., as suggested by CT scan or recruitment to inflation ratio) [87]. In low recruitable lungs, PEEP above a certain level might be harmful by increasing strain and dead space (increased PCO2) resulting in severe lung injury and deleterious hemodynamic effects, leading to reduced net oxygen delivery [88,89].

## 12. Therapeutic Approaches in the ED

For asymptomatic COVID-19 patients, no special treatment is needed, other than domestic isolation, adequate nutrition and hydration, and medical observation for a certain amount of time according to national guidelines and local protocols. For appropriate contact tracing, epidemiological, demographic data must be collected, and patients should be given medical advice to be reexamined in case of late onset related symptoms of COVID-19 [9].

For those who will be hospitalized or admitted to the ICU due to respiratory failure, supportive care is the mainstay of treatment as severe COVID-19 is a significant cause of morbidity and mortality and its diagnosis and management is challenging in the ED [90]. Confirmed cases should have close hemodynamic monitoring, mainly the elderly or those with co-morbidities such as patients with coronary heart disease, chronic respiratory diseases, hypertension, diabetes, chronic renal diseases, etc. [91].

The antiviral agent remdesivir, a prodrug of adenosine nucleotide analog, provides a certain broad-spectrum antiviral activity against several RNA viruses and was associated with improved time to recovery in patients who required oxygen supplementation but not high flow oxygen, non-invasive ventilation, or invasive mechanical ventilation [92]. Thus, it is recommended for patients who require minimal supplemental oxygen. Furthermore, its benefit appears to be greater when given earlier in the illness [93].

Systemic corticosteroids, as anti-inflammatory agents, are recommended for COVID-19 patients who require any type of respiratory support including mechanical ventilation during their ED stay [94]. Treatment with 6 mg of dexamethasone per day for up to 10 days to reduce cytokine-related pulmonary damage in patients with COVID-19 pneumonia has been shown to reduce mortality at 28 days [95]. The benefit of dexamethasone was greater in patients who required more respiratory support and is therefore recommended for patients who require increasing amounts of supplemental oxygen. If dexamethasone is not available an equivalent dose of another corticosteroid may be used. The combination of corticosteroids and remdesivir is preferred to corticosteroid monotherapy in those patients because corticosteroids might slow viral clearance when administered without an antiviral drug [96].

In patients with rapid respiratory deterioration who require oxygen support with high flow device or noninvasive ventilation and have increased markers of inflammation, the addition of tocilizumab, an interleukin-6 receptor antagonist to corticosteroids, has been shown to reduce mortality in two large randomized controlled studies [97,98]. If tocilizumab is not available, sarilumab can be used in combination with corticosteroids. Moreover, the combination of tocilizumab with corticosteroids is recommended in patients that require mechanical ventilation.

In patients with severe or critical illness there is not yet sufficient evidence to support or reject the administration of empiric broad spectrum antimicrobial therapy. However, if bacterial coinfection is suspected, antimicrobials should be administered but reassessed daily to minimize adverse consequences [99,100].

Thromboembolic risk and anticoagulant therapy in COVID-19 patients is another therapeutical option that emergency physicians should be take into consideration [101]. The CHEST guidelines [102] and the ISTH guidelines [103] both suggest the use of standard dose anticoagulant thromboprophylaxis over intermediate- or full-dose anticoagulation.

## 13. Monoclonal Antibody Infusion in the ED

Recently, the U.S. Food and Drug Administration (FDA) issued an Emergency Use Authorization (EUA) for two cocktails of monoclonal antibodies (mAbs); casirivimab/imdevimab (REGN-COV2) and bamlanivimab/etesevimab [104]. These mAbs have been authorized for use in non-hospitalized patients, 12 years of age or older, weighing at least 40 kg, with mild to moderate COVID-19 but at high risk of disease progression and/or hospitalization. This includes those who are 65 years of age or older or who have certain chronic medical conditions.

Casirivimab and imdevimab, used in a combined cocktail called REGN-COV2, are administered together intravenously. Both mAbs bind to non-overlapping epitopes of the SARS-CoV-2 S protein receptor-binding domain and potently neutralize the entry of the virus into the host cells. A significant reduction in viral load was demonstrated when this mAbs cocktail was administered in 182 symptomatic non-hospitalized COVID-19 patients versus 93 patients that received a placebo. Safety outcomes were similar in the REGN-COV2 and placebo group. Moreover, REGN-COV2 reduced COVID-19 related hospitalizations or emergency room visits within 28 days after treatment, when compared to the placebo [105].

Bamlanivimab and etesevimab bind to different, but overlapping, epitopes within the receptor-binding domain of the SARS-CoV-2 S protein and block the entry of the virus into the host cells. When 2800 mg of bamlanivimab and 2800 mg of etesevimab were given to 112 non-hospitalized patients with mild to moderate COVID-19 symptoms, the viral load reduction was statistically significant at day 11 compared with the placebo group (156 patients). On the other hand, bamlanivimab monotherapy showed no significant improvements in terms of viral load reduction [106]. Furthermore, among 1035 high-risk ambulatory patients, bamlanivimab plus etesevimab led to a lower incidence of COVID-19 related hospitalization and death than the placebo, and accelerated the decline in the SARS-CoV-2 viral load [107].

Ideally, mAbs should be given in designated infusion centers; however, such centers do not exist in all hospitals. EDs can be used to provide these infusions with safety, but only if adequate space and staff exist to ensure minimal impact on usual ED care and flow. A real-life study demonstrated a significant length of stay associated with REGN-COV2 infusion in the ED (477 min), so attention should be paid to this [108].

## 14. Impact of Vaccination on ED Care

As soon as the FDA issued an EUA for the Pfizer-BioNTech COVID-19 vaccine, the U.S. began a nationwide vaccination campaign. The CDC reported that compared with the prevaccination period (29 November–12 December 2020), in the postvaccination period (18 April–1 May 2021) COVID-19 ED visits per 100,000 ED visits declined by 59% among all adults, especially for persons aged ≥65 years (77%) [109]. The greater decline in older adults, who had higher vaccination coverage compared to younger adults, provides evidence of the likely contribution of vaccination coverage to reducing ED visits.

A significant decrease in hospitalizations for all age groups was also shown by an Israeli study, as cumulative vaccination coverage increased during the first 4 months of the nationwide vaccination campaign [110]. These findings suggest that COVID-19 vaccination can help to control the pandemic and decrease ED visits.

## 15. ED-Based COVID-19 Vaccination

EDs see more than 150 million patients per year in the U.S. and often serve vulnerable populations who may lack primary care and have suffered disproportionate COVID-19 pandemic effects [111]. For this reason, EDs represent an important public health opportunity for COVID-19 vaccination programs. Furthermore, healthcare providers are the most trusted source of health information and can thus build confidence and improve COVID-19 vaccine uptake [112].

Each site has to decide whether to and who to vaccinate, taking into consideration local resources, local demand, and the ability to refer patients for their second vaccination [113]. The overall priority is to offer vaccination to all eligible patients, emphasizing on vulnerable populations to whom the ED has unique access like immigrants and limited English-language proficiency communities, low-income populations, communities of color, and other underserved populations.

The COVID-19 ED-based vaccination program should take place at the triage, during treatment, or during hospital discharge. However, it should be kept in mind that ED-based vaccination should fit within the normal ED flow. Low volume EDs may not be able to sustain an effective program. On the other hand, the ED may be unable to provide vaccination during times of extremely high volume that strain available resources. While modifications may be necessary, they should not lead to extended lengths of stay nor have negative impact on usual ED care. Thus, the dual focus of this program should be on ensuring high rates of vaccination and minimal impact on usual ED care and flow.

Since it is hard to predict when the supply chain and procurement processes will allow for distribution to EDs, EDs should have a certain degree of flexibility and readiness in this process.

## 16. Evidence Based Recommendations

Evidence-based practice in the approach of COVID-19 is necessary but keeping up with ever-changing information is difficult. Table 2 summarizes the evidence-based recommendations for the management of COVID-19 patients in the ED.

## 17. Conclusions

In conclusion, the management of this new and unexpected pandemic requires a careful reorganization of the EDs. The initial management of COVID-19 patients requires high and rapid clinical suspicion and validated and well-timed triage and risk stratification. The priority is to minimize virus transmission, to provide high quality healthcare services, to reduce lengthy waiting times, and to enhance the overall outcome among patients with COVID-19 disease. Our paper provides updated, evidence-based recommendations for the management of COVID-19 patients in the ED.

## Figures and Tables

**Table 1:** Performance of deterioration risk scores in COVID-19 patients.

First Author	Triaging Test	Population/Study Type	Outcome	Discriminatory Performance (ROC AUC)
Beals et al. [13]	Rothman index	3499 COVID-19 patients (retrospective, multicenter study)	Identification of patients at admission with high risk for subsequent deterioration	0.81–0.84
Su et al. [14]	CRB-65, qSOFA	116 COVID-19 patients (retrospective single center study)	Identification of patients who require intensive respiratory or vasopressor support	0.81 ± 0.05 for CRB-65 0.70 ± 0.06 for qSOFA
Volff et al. [15]	NEWS, mNEWS	363 COVID-19 patients (retrospective single center study)	Identification of patients at risk for clinical deterioration (ICU admission or death)	0.74 for NEWS 0.72 for mNEWS
Guo et al. [16]	CURB-65	74 COVID-19 patients (retrospective single center study)	Identification of patients at risk for in-hospital death	0.81
Hu et al. [17]	MEWS, REMS	105 COVID-19 patients (retrospective single center study)	Identification of patients at risk for in-hospital death	0.677 for MEWS 0.833 for REMS
Ucan et al. [18]	PSI, CURB-65 A-DROP	298 patients with probable or definitive COVID-19 (retrospective single center study)	Identification of patients at risk for in-hospital death and progression to severe disease	PSI: 0.873 for overall mortality & 0.697 for progression to severe COVID-19 CURB-65: 0.859 for overall mortality & 0.739 for progression to severe COVID-19 A-DROP: 0.875 for overall mortality & 0.660 for progression to severe COVID-19

ROC AUC: Area Under the Receiver Operating Characteristic Curve, qSOFA: quick Sequential Organ Failure Assessment, NEWS: National Early Warning Score, mNEWS: modified National Early Warning Score, MEWS: Modified Early Warning Score, REMS: Rapid Emergency Medicine Score, PSI: pneumonia severity index.

**Table 2:** Evidence based recommendations for management of COVID-19 patients in the ED.

**Triage**	Screen patients for possible COVID-19 symptoms Suspected COVID-19 patients should not be mixed with COVID-19 confirmed patients in isolation areas Manage the risk classification for disease severity ideally in a prehospital isobox facility Critically ill patients should be transferred to the ICU as soon as possible with no delay The priority is to minimize virus transmission
**Illness severity**	Distinguish between mild, moderate and severe illness (see text) Patients with moderate or severe illness should be admitted to the hospital and closely monitored Be aware of ICU transmission for critically ill patients
**Deterioration risk prediction**	Effective triage is crucial for informing clinical decision making and facilitating resource allocation The 4C (Coronavirus Clinical Characterisation Consortium) Mortality Score and the 4C Deterioration Score could provide an evidence-based method to identify those who will need aggressive support during admission, even if they have a low risk of death
**Imaging**	Chest CT is not routinely recommended in asymptomatic patients or in those with mild symptoms Chest CT plays a key role in the diagnosis of COVID-19 and monitoring of disease progression Bilateral and multilobar infiltrations are associated with poor prognosis CT angiography is indicated if pulmonary embolism is suspected Lung ultrasound (US) may play a complementary role because of its advantages over the use of chest CT (radiation-free, flexibility, and cost-effective) Lung US may be considered as a safe and alternative to chest CT imaging method for pregnant women as it is radiation-free Lung US maybe useful to identify alternative pathologies resulting in respiratory failure
**Laboratory tests**	Asymptomatic patients or those with mild disease should not routinely undergo laboratory blood tests Patients with moderate, severe, or critical COVID-19 infection are recommended to be tested with complete blood count, biochemical tests, coagulation markers, and inflammatory markers Thrombocytopenia, neutropenia, new acute kidney injury, elevated transaminases, ferritin levels, troponin, lactate dehydrogenase, d-dimers, and CRP are associated with worse disease outcome
**Intubation**	The best timing for endotracheal intubation (early vs. late) remains a controversial topic Time of intubation may have no effect on the all-cause mortality and morbidity of critically ill patients with COVID-19 A trial with high flow nasal or non-invasive ventilation is recommended in critically ill patients with COVID-19 before intubation The decision to intubate involves judgement and should be individualized and based not only on the oxygenation status but also on the degree of respiratory distress
**Therapeutic approaches**	For asymptomatic COVID-19 patients, no special treatment is needed or recommended. Domestic isolation, adequate nutrition and hydration, and medical observation according to national guidelines and local protocols are recommended For those hospitalized or admitted to ICU due to respiratory failure, supportive care is the mainstay of treatment Remdesivir is recommended in patients who require minimal supplemental oxygen, early in the disease course Systemic corticosteroids are recommended for COVID-19 patients who require any type of respiratory support including mechanical ventilation In patients with rapid respiratory deterioration who require oxygen support with high flow device or non-invasive ventilation and have increased markers of inflammation, the addition of tocilizumab is recommended Anticoagulant thromboprophylaxis in COVID-19 patients is indicated. The use of standard dose over intermediate or full dose is preferred if pulmonary embolism is not confirmed or highly suspected If bacterial coinfection is suspected antimicrobials should be administered but reassessed daily to minimize adverse consequences of unnecessary antibiotic therapy The newly approved cocktails of monoclonal antibodies (casirivimab/imdevimab and bamlanivimab/etesevimab) may have a potential role in specific patients

## Data Availability

Not applicable.
